# Variations in older people’s use of general practitioner consultations and the relationship with mortality rate in Vantaa, Finland in 2003–2014

**DOI:** 10.1080/02813432.2019.1684426

**Published:** 2019-11-11

**Authors:** Katri Mustonen, Timo Kauppila, Ossi Rahkonen, Jarmo Kantonen, Marko Raina, Tiina Mäki, Kaisu Pitkälä

**Affiliations:** a Department of General Practice and Primary Health Care, Faculty of Medicine, University of Helsinki, Finland;; b Department of Public Health, Faculty of Medicine, University of Helsinki, Finland;; c Health Care and Social Services, City of Vantaa, Finland;; d Attendo LDT, Helsinki, Finland;; e Oy Apotti Ab, Helsinki, Finland;; f HUSLAB Laboratory Services, Helsinki University Hospital, Helsinki, Finland;; g Helsinki University Hospital, Unit of Primary Health Care, Helsinki, Finland

**Keywords:** Epidemiology, older adults, health services research, family practice, gender

## Abstract

**Objective:** It is generally expected that the growth of the older population will lead to an increase in the use of health care services. The aim was to examine the changes in the number of visits made to general practitioners (GP) by the older age groups, and whether such changes were associated with changes in mortality rates.

**Design and setting:** A register-based observational study in a Finnish city where a significant increase in the older population took place from 2003 to 2014. The number of GP visits made by the older population was calculated, the visits per person per year in two-year series, together with respective mortality rates.

**Subjects:** The study population consisted of inhabitants aged 65 years and older (65+) in Vantaa that visited a GP in primary health care.

**Main outcome measures:** The number of GP visits per person per year in the whole older population during the study years.

**Results:** In 2009–2010, there was a sudden drop in GP visits per person in the younger (65–74 years) age groups examined. In the population aged 85+, use of GP visits remained at a fairly constant level. The mortality rate decreased until the year 2008. After that, the positive trend ended and the mortality rate plateaued.

**Conclusions:** Simultaneously with the decline in GP visits per person in the older population, the mortality rate leveled off from its positive trend in 2009–2010. Factors identified being associated with the number of GP consultations were organizational changes in primary health care, economic recession causing retrenchment, and even vaccinations during the swine flu epidemic.

**Key points**
- Along with an increasingly ageing population, concern over the supply of publicly funded health care has become more pronounced.
- The amount of GP visits of 65+ decreased in primary health care, especially in the youngest groups.
- However, in the oldest age groups (85+), the use of GPs remained unchanged regardless of changes in service supply.
- As the rate of GP visits among the population of 65+ declined, the positive trend in the mortality rate ceased.

## Background

Health care use and expenditures will change as the baby boomers approach old age [1]. Some scenarios suggest that expenditure will grow due to older people using health care services more [1–3]. Other scenarios assume that age is not the main factor in health care expenditure, but rather time-to-death [4,5]. In a Finnish study, a positive relationship was found between age and higher use of primary health care services among those not being treated in long-term care [6]. There are indications that the ageing population will require increased resources [7], in office-hour medical services [8–11] and emergency medical services [12].

Finland, as a developed country, faces the challenge of a rapidly increasing older population [13]. Health care is organized by municipalities in Finland. Thus, resources allocated to health care services differ according to the economic balance of the local communities and their general interest in investing in health care. During the last 20 years specialized care has received investments, whereas the development and growth of primary health care has been more modest. In the period from 1997 to 2005, for the whole of Finland, there was a constant decrease in the number of visits to GPs in health centers per inhabitant over 65 years of age per year: from 4.3 (in 1997) to 3.2 visits per inhabitant (in 2005) [14], whereas in 2009 the same age group had only a mean of 2.4 GP visits per year [15]. In a Swedish study, over-65-year-old men had an average of 3.4–3.8 GP visits per year and women 4.2–4.5 GP visits per year in 2007–2011 [16]. The official statistics of Finnish health care services revealed that in 2001 the total number of visits to primary health services was 25,000,000. Of these, doctors’ appointments constituted 40% and visits to other health care professionals (mainly nurses) 60%. Over the years, the proportion of doctors’ appointments has steadily diminished, in 2010 being less than 30% of all visits to primary care health centers [17].

Health services can be considered from the aspects of supply, demand, and need [18]. Supply is the actual provision of services, and it is regulated by health officials. Demand refers to how much citizens seek services, and it can be measured as, for example, waiting times for doctors' appointments. Responding to demand may not always lead to increased health benefits. Need may be defined as a population’s objectively measured health needs, the meeting of which will presumably lead to health benefits. Information concerning the need for care is not directly obtainable from any one source, but requires indicators from indirect sources such as morbidity, mortality, or combinations of abilities of working and functioning [19]. Objective health needs are difficult to define or measure. Since objective information on need is difficult to acquire, follow-up should consist of a wide range of outcome measures and different indicators, reflecting also unpredictable consequences [18]. Apart from health problems and effective treatments, needs are also affected by a variety of other variables such as demographic factors, people’s values and attitudes towards the use of services, and proximity and availability of services [19]. While use depends on both demand and supply, the objective need is something that may often only be evaluated afterwards.

Vantaa is a typical Finnish city where a strong increase in the older population took place in the early 2000s. This has presumably had an effect on the demand and need for health care services. During the years 2003–2014 the number of 65+ in Vantaa had almost doubled from 16,871 to 30,540, accounting for 9% of the entire population of Vantaa in 2003, and 15% in 2014. This age group often visits GP due to cardiovascular or endocrine diseases, where the GP has an important role [20]. According to a European study, the likelihood of multi-morbidity increased with age, and the incidence of chronic conditions led to greater health care use in both primary and secondary care [21].

During the study period Vantaa underwent changes in its organization of primary health care services, which affected the supply of GP appointments for older citizens. In 2007, Vantaa re-arranged the structure of all of its primary health care services to a hierarchical form. In 2008, a new geriatric unit was established for multi-morbid older patients needing comprehensive geriatric assessment and rehabilitation, and the same year the whole primary care emergency unit was outsourced [22]. The following year (2009), there was a worldwide pandemic of A(H1N1)-virus, swine flu, in Finland that reduced the supply of slots for GP visits due to a large vaccination campaign. It affected Vantaa’s health care processes. Since complications were expected, GPs were involved in the process as overseers, leading to a 10% reduction in GP visits relative to 2008 whereas nurses’ visits remained at approximately the same level as in the preceding year [23].

As health care services in Finland are funded by taxes, economic booms and recessions also affect the supply of services. During the study period there was a global financial crisis in the year 2007, causing a recession also in Finland from 2008 onwards. In 2009, this economic recession caused retrenchments in Vantaa’s public services [23].

### Aims

The aim was to examine the changes in GP visits in the increased age groups of 65+, in Vantaa primary health care in 2003–2014. In addition, a further aim was to examine the associations between the number of GP visits and mortality rates.

## Methods

### Setting

This is an observational and descriptive study of the primary care of the third largest city of Finland (Vantaa with 210,000 inhabitants in 2014). Vantaa is located northeast of Helsinki, the capital city. Older people’s use of GP consultations in public health care during 2003–2014 was retrospectively explored. The study population comprises population of 65+ in Vantaa. Public primary care in Finland is practically free of charge, which makes it popular among the retired population. Both the total crude numbers and the visit rate ratios for males and females were separately calculated. All data were gathered and analyzed in a manner that allowed patients’ and doctors’ anonymity to be maintained. The health authorities of the City of Vantaa and the Ethics Board of the City of Vantaa (TUTKE) approved the study protocol (VD/8059/13.00.00/2016).

The GP visit data were obtained from the electronic health records of the City of Vantaa (Graphic Finstar – patient chart system, Logica Ltd., Helsinki, Finland). It consists of GP visits during office hours and in the evening in primary care. Treatment in primary care wards or long-term care is not included in the data. Mortality data were gathered from the official statistics provided by Statistics Finland. The data obtained from these sources were divided into the age groups of 65–69, 70–74, 75–79, 80–84, and ≥85 years. The number of GPs working in primary care was extracted from the files of Social and Health Services of the City of Vantaa.

Visit rates (per 1000 persons per year) with 95% confidence intervals (CIs) were calculated assuming a Poisson distribution. Poisson rate regression was performed to test for significant trends in visit rates and mortality across calendar years. The Poisson regression model was tested using a goodness-of-fit test, and assumptions of over-dispersion in the Poisson model were tested using the Lagrange multiplier test. All analyses were performed using STATA software, version 15.1 (StataCorp LP, College Station, TX, USA).

### Statistical methods

In order to segregate outpatients from inpatients, the inpatients were excluded from the data. The remaining data were evaluated by using two-way analysis of variance and then assessed for credibility. The study examined how many GP visits 65+ had annually in health centers and how these numbers changed over time (from year 2003 to 2014). Mortality rates in Vantaa primary care were collected for the whole investigation period.

## Results

**Figure 1.**
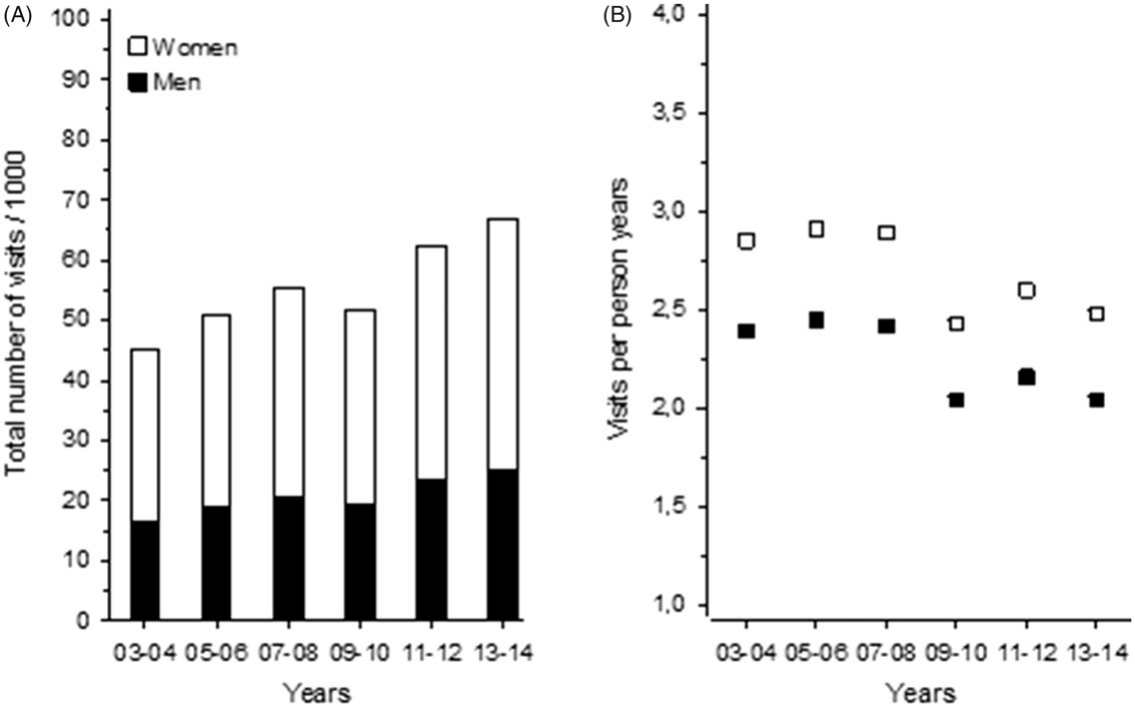
(A) Total number of GP visits per 1000 65+ year-old inhabitants in Vantaa during 2003–2014. (B) Total visits divided per person per year, describing how many visits one 65+ has per year on average in Vantaa in during 2003–2014.

The total number of GP visits 65+ increased steadily during the study period, from 2003 to 2014 (Figure 1A). The only difference was seen in 2009–2010, when the number of GP visits slightly declined. On the other hand, when calculated per person per year in the whole older population, there was a significant relative decrease in GP visits during the same years 2009–2010, and this decrease was sustained over the follow-up (Figure 1B). There was no gender difference in this phenomenon.

**Figure 2.**
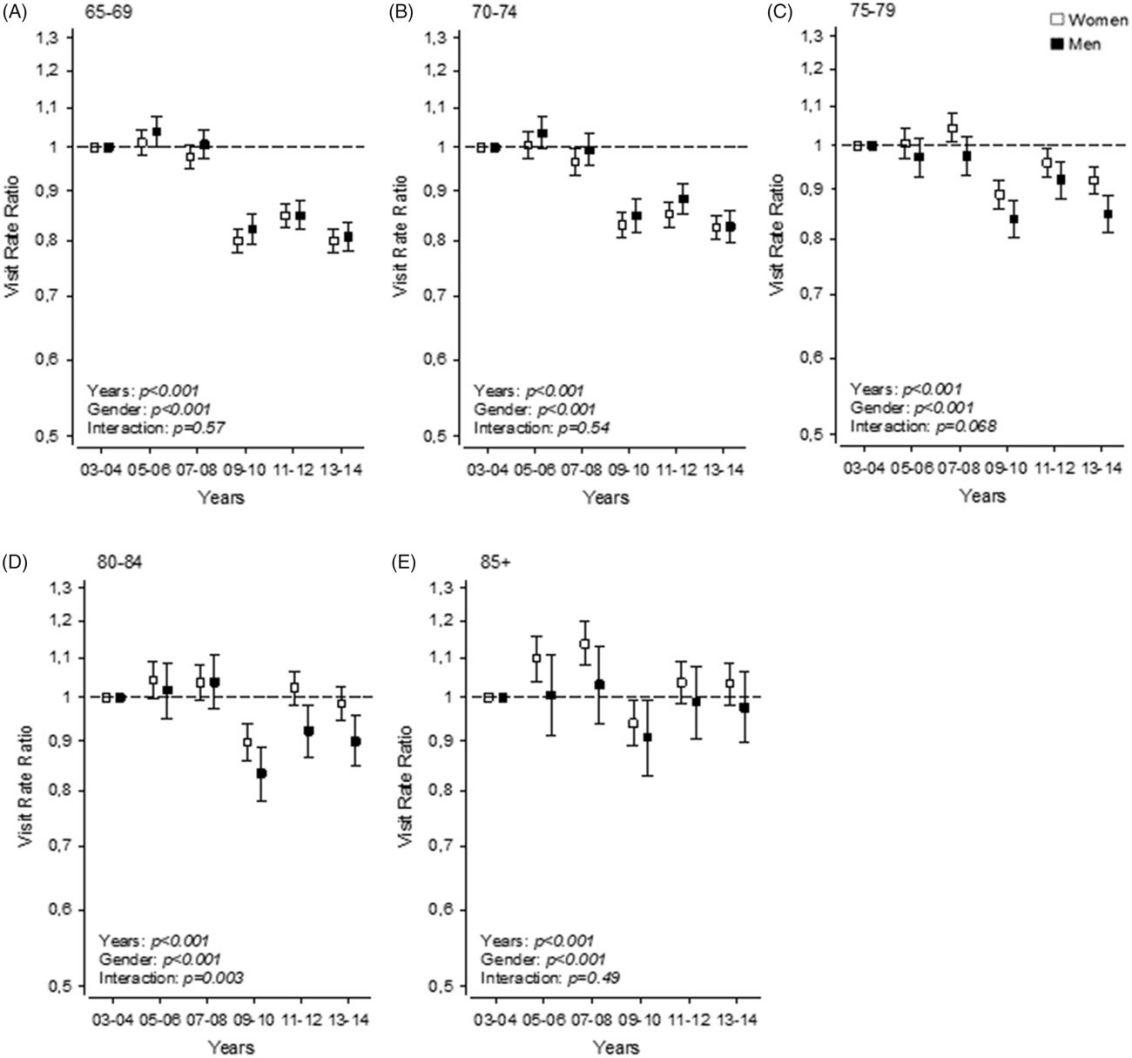
Visit rate ratios of GP visits in the years 2003–2014: (A) age group of 65–69 year-olds, (B) age group of 70–74 year-olds, (C) age group of 75–79 year-olds, (D) age group of 80–84 year-olds, (E) age group of ≥85 year-olds. The years 2003–2004 are used as reference and 95% confidence intervals are shown with brackets.

This change was not consistent in all age groups. In the youngest age group of 65–69 years, the visit rate ratio remained at the 2003–2004 level (rate ratio reference), until 2009–2010, when it dropped 20% and stayed at this level (Figure 2A). A similar trend was seen in the age group 70–74 years (Figure 2B). In the age group 75–79 years, the visit rate ratio remained at the 2003–2004 level, until 2009–2010, when it dropped about 10%, remaining slightly below the 2003–2008 level (Figure 2C). In the age group 80–84 years, a similar pattern was seen; the visit rate ratio stayed at the same level until 2008, after which it decreased by approximately 10-15% in both sexes. From the year 2011 onwards, this ratio increased back to the starting level in women (Figure 2D), but not in men. In all age groups, women’s use of GP visits was higher than men’s use. In the age group ≥85 years, the visit rate ratio changed less than in the other age groups. From 2005 onwards, the visit rate ratio increased 10% in the women’s group, but remained the same in the men’s group, until 2009–2010, when a decrease below the 2003–2004 level was seen on both sexes (Figure 2E). After this, the level in both sexes returned to the starting level.

**Figure 3.**
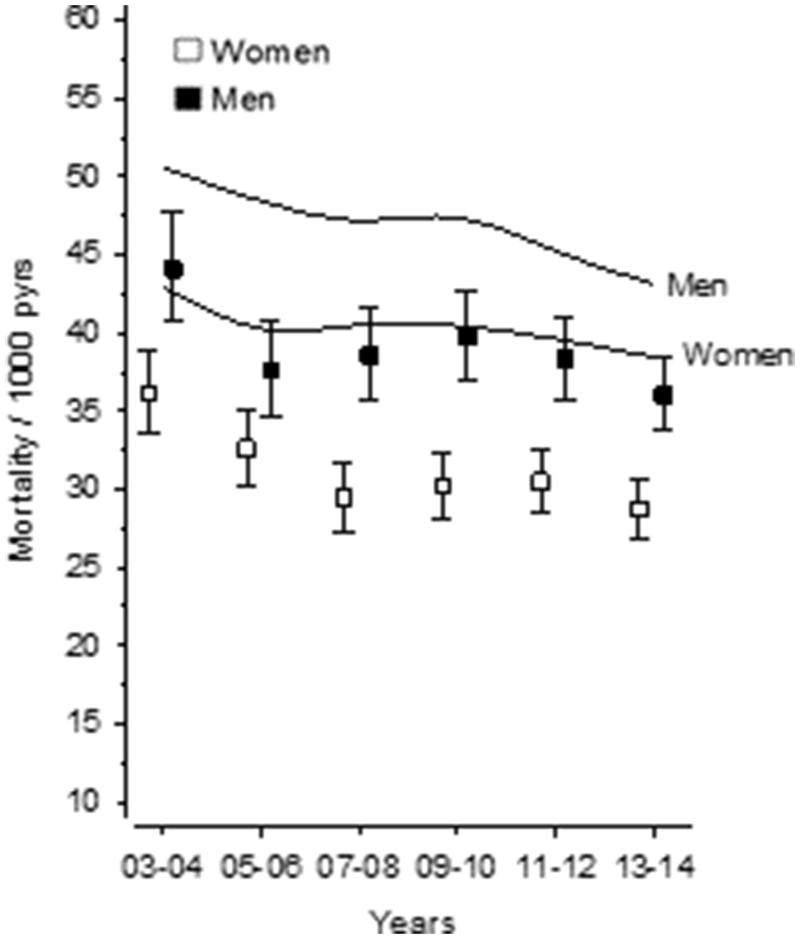
Mortality rate per 1000 persons per year of 65+ in the whole of Finland (curves indicating men and women) compared to mortality rate per 1000 persons per year of 65+ in Vantaa during 2003–2014.

When investigating the mortality rate per 1000 persons per year in Vantaa over the same period, there was an overall decreasing trend. However, this positive trend seemed to level off in 2007–2012 in men and in 2009–2012 in women. Similar trends in mortality rate were observed throughout Finland over the same period. Men’s mortality rate was higher than women’s during the whole time. When Vantaa was compared with Finland overall, both men’s and women’s mortality rates were lower in Vantaa (Figure 3).

## Discussion

This paper analyzed the use of GP visits and changes in GP visits and mortality ratio 65+ during a 12-year period in a large Finnish city. The most remarkable observation was that in 2009–2010 in all older age groups the visit rate ratio decreased suddenly after a long period of relative stability. In the younger age groups, the level remained lower thereafter, whereas in the oldest population groups (80 years and over) the rate returned to the original level or surpassed it in 2011–2014. Changes were also observed in the mortality rate, with a pause occurring in the decreasing trend in 2009–2012 for both men and women, until 2013–2014, when the mortality rate resumed its decreasing trend.

These findings may indicate that the supply of health care GP consultations was restricted from 2009 onwards, and the oldest age groups had a larger share of the restricted GP appointments. There may be several explanations for the decrease in GP consultations. Firstly, the swine flu epidemic in 2009 had an effect on health care resources in Vantaa, as nurses were busy vaccinating patients and GPs monitored the vaccinated patients [23], but this effect was short-lived. Secondly, nurse’s appointments became more frequent at the same time, changing the structure of outpatient consultations. Thirdly, the financial crisis began in 2007 globally, causing a recession in Finland from 2008 onwards, which had effects on public service budgets planned for 2009. Savings policies were implemented also in Vantaa.

The mortality rate was also investigated, as it represents an outcome reflecting the objective health need of older population. The favorable trend in decreasing mortality appeared to pause in 2009–2010 (–2012), corresponding to the decrease in the supply of GP consultations. This is in line with an Icelandic study suggesting that regular GP visits maintained patients’ longevity in several chronic diseases since appointments were important for recognizing symptom escalation, allowing treatment to be adjusted before the occurrence of more serious events [24]. It is also in line with a recent American study showing that the supply of GPs is associated with mortality [25]. There may also be other explanations for the changing mortality trends. The swine flu epidemic might have caused increased mortality in 2009 in older age groups. This is supported by a similar phenomenon in mortality simultaneously occurring for the entire Finnish population. The mean mortality rate of individuals aged 65+ years in Vantaa was at a modest level relative to other parts of the country.

In certain chronic conditions, such as heart failure and diabetes, it has been suggested that the chronic care model with a nurse-managed program decreases mortality [26,27]. However, a systematic review including 33 studies concluded that there is insufficient evidence for a beneficial effect of the chronic care model on mortality of multi-morbid patients [28]. Moreover, a Cochrane review from the year 2005 suggested that shifting tasks from doctors to nurses did not change the treatment processes, the use of resources, or the costs. In general, the nurses provided more health advice to patients, therefore achieving a higher patient satisfaction rate, but they also ordered more additional services and tests, diminishing the cost benefit obtained from shifting tasks from doctors [29]. A Finnish-Norwegian study found that in Finland more individuals aged 65–74 years used specialists’ services than in Norway. In Norway, the same-aged population more often visited GPs. This difference might be a sign of either stronger gate-keeping in Norway or overflow from the Finnish public system to the private sector or to specialized care [30]. This might reflect an increased demand that the Finnish public sector cannot meet. Examining the demands of health care, i.e. the waiting times or queues to GPs and the overflow to the private sector or to specialized care, was beyond the scope of this study.

### Study strengths and limitations

The strength of this study lies in the long investigation period, with valid register data, reflecting true changes in GP consultations over time. Research of the changes in GP consultations simultaneously with the mortality ratio has not been conducted before and therefore, this study provides novel information. The mortality rates are based on Finnish population registers, which are 100% complete.

The main limitation of this study is that the real reasons behind the changes in GP appointment supply are difficult to examine. In addition, this paper does not have information regarding other health services, i.e. private sector or specialized services in hospitals that the study population used during the study period.

## Conclusions

The results indicate that in Vantaa during 2003–2014 the older population’s GP visits decreased, when calculated per person per year, as the total number of older people increased. Simultaneously with the decline in GP visits per person in the ageing population, the mortality rate leveled off its positive trend in 2009–2010. A single definitive reason for the changes in 2009 cannot be identified. Factors found to affect the supply of GP consultations include changes in organization of the service system in primary health care, economic recession causing retrenchment, and vaccinations during the swine flu epidemic. The approach to examining health care supply, its actual use, and a surrogate variable for health care needs – mortality – reveals interesting simultaneous trends and phenomena in these variables that may be connected to organizational changes in health care, society-level economics, and the effects of epidemics.
